# The C228T mutation of TERT promoter frequently occurs in bladder cancer stem cells and contributes to tumorigenesis of bladder cancer

**DOI:** 10.18632/oncotarget.4295

**Published:** 2015-06-17

**Authors:** Chong Li, Song Wu, Haifeng Wang, Xingang Bi, Zhao Yang, Ying Du, Luyun He, Zhiming Cai, Jiansong Wang, Zusen Fan

**Affiliations:** ^1^ CAS Key Laboratory of Infection and Immunity, Institute of Biophysics, Chinese Academy of Sciences, Beijing 100101, China; ^2^ Department of Urology, The Second People's Hospital of Shenzhen, Shenzhen 518035, China; ^3^ Department of Urology, The Second Affiliated Hospital of Kunming Medical College, Kunming 650101, China; ^4^ Cancer Institute & Hospital Chinese Academy of Medical Sciences, Beijing 100021, China

**Keywords:** bladder cancer stem cells, C228T mutation, telomerase reverse transcriptase (TERT), tumorigenesis

## Abstract

Bladder cancer is one of the most common malignant tumors worldwide. Bladder cancer stem cells (BCSCs) have been isolated recently but have not been defined yet. Here we sorted BCSCs from bladder tumor tissues or normal bladder stem cells (NBBCs) from adjacent normal bladder tissues. We found that the C228T mutation (chr5, 1, 295, 228 C > T) of TERT promoter frequently occurs in BCSCs, but not exist in NBBCs. Importantly, introducing the C228T mutation in NBBCs causes TERT overexpression and transformation of bladder cancer. Restoration of the C228T mutation to T228C in BCSCs can recover the TERT expression to a basal level and abolish tumor formation. Additionally, the C228T mutation of TERT promoter triggers TERT expression leading to increased telomerase activity. TERT expression levels are consistent with clinical severity and prognosis of bladder cancer.

## INTRODUCTION

Bladder cancer is one of the most common malignancies, ranking the fifth most common cancer in Western society [1, 2]. The majority of bladder cancers are histologically classified as urothelial carcinomas [3]. Urothelial carcinomas originate from the bladder urothelium consisting of basal, intermediate, and umbrella cells, representing early, mid, and later differentiation states, respectively [4]. Malignant transformation can occur in any of these cell types that gives rise to tumors with diverse phenotypes [5]. Over 70% is non-muscle-invasive tumors, whereas the remainder is muscle-invasive tumors [6]. However, the tumorigenesis of bladder cancer remains elusive.

The cancer stem cell (CSC) hypothesis is relied on the notion that cancers are maintained by a rare subpopulation of self-renewing tumor-initiating cells (TICs) [7, 8]. The CSCs possess stem cell properties such as self-renewal and differentiation. A small subset of CSCs accounts for sustaining tumorigenesis and forming the cellular heterogeneity of tumors. CSCs have been demonstrated to occur in many solid tumors [9]. Recently, bladder CSCs (BCSCs) were identified, whose signature surface makers are similar to those of normal bladder basal cells (NBBCs) [10, 11]. It is strongly needed to get insights into the molecular alterations in CSCs for development of new therapies.

Immortalization of cancer cells is one of the cancerous hallmarks, which requires to counteract telomeres that shorten at each round of DNA replication. The major telomere maintenance needs the telomerase that synthesizes de novo telomeres [12]. Telomerase is composed of two subunits: telomerase RNA template (TERC), and telomerase reverse transcriptase (TERT) catalytic subunit [13]. Analyses of whole-genome sequencing data from malignant melanomas revealed two somatic TERT gene promoter mutations [14]. These mutations form de novo consensus binding motifs for E-twenty-six (ETS) transcription factors, leading to TERT activation. Recent studies have reported that TERT is implicated in the modulation of cancer stem cells [15, 16]. Here we show that the C228T mutation of TERT promoter highly occurs in bladder cancer stem cells and this mutation contributes to tumorigenesis of bladder cancer.

## RESULTS

### The C228T mutation of TERT promoter appears in BCSCs with high frequency

**Figure 1:**
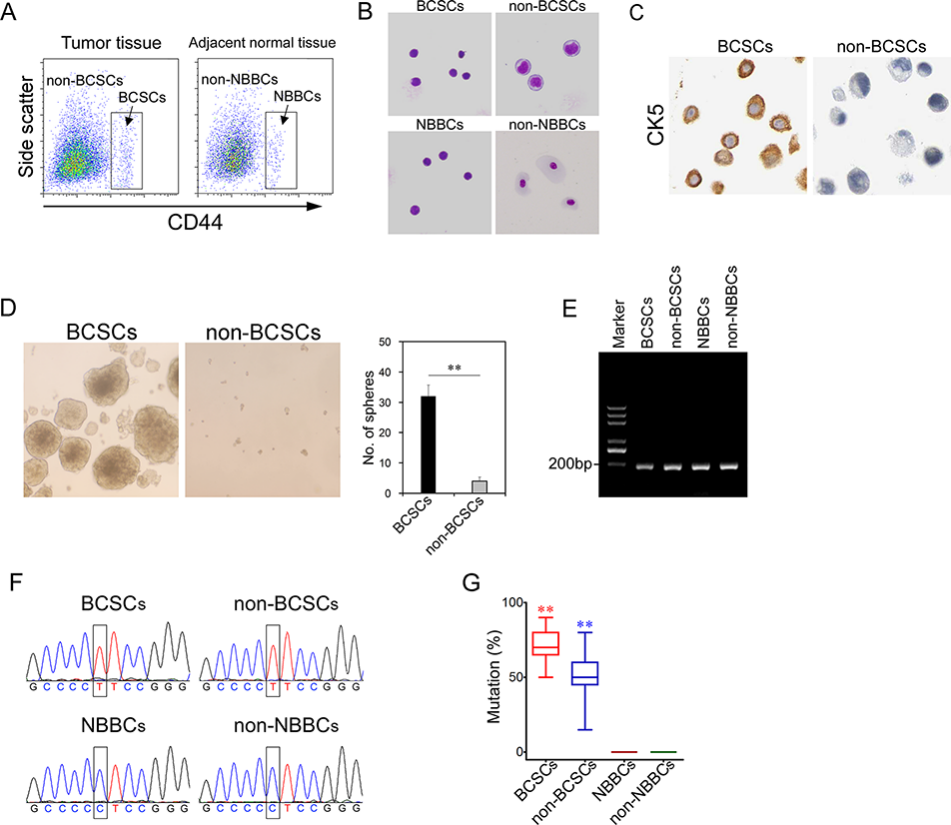
The C228T mutation of TERT promoter frequently occurs in BCSCs **A.** BCSCs or NBBCs were sorted with CD44 from bladder tumor tissues or adjacent normal bladder tissues by flow cytometry. **B.** Cellular morphology of fractionated cells was observed by Giemsa-Wright staining. **C.** BCSCs and non-BCSCs were stained by anti-CK5 antibody. **D.** Sphere formation assay shows BCSCs (left panel) displayed significantly enhanced growth capability than non-BCSCs (right panel). **E.** Single cell genomic DNA was extracted and performed single-cell PCR with specific primers for the TERT promoter. **F.** Sequence chromatograms of fractionated cells by Sanger DNA sequencing. **G.** The mutation rates of fractionated cells were presented. **p < 0.01.

BCSCs were recently isolated in bladder cancer samples with the surface makers (Lineage-CD44+CK5+CK20-) [10], similar to normal bladder basal cells (NBBSc). Several recent studies showed that TERT core promoter mutations occur in melanoma and other cancers [17–19]. We wanted to determine whether TERT core promoter mutations occur in BCSCs. We collected freshly isolated bladder cancer specimens from sixty patients (Supplementary Table 1). We sorted BCSCs and non-BCSCs from bladder tumor tissues through flow cytometry with CD44 (Figure 1A). Simultaneously, NBBCs and non-NBBCs were isolated from adjacent bladder normal tissues with the same surface marker. Cellular morphology of BCSCs, non-BCSCs, NBBCs and non-NBBCs were prepared by cytospinning followed by Giemsa-Wright staining (Figure 1B). BCSCs and NBBCs possessed a high nuclear to cytoplasmic ratio, relatively smaller and homogenous in size. The morphology of BCSCs was similar to that of NBBCs. In contrast, non-BCSCs were characterized by tumorous features (Figure 1B). Non-NBBCs were characteristic of differentiated cells. Moreover, CK5 was stained positive on BCSCs (Figure 1C). BCSCs exhibited significantly higher oncosphere-forming efficiency compared with non-BCSCs (Figure 1D).

We extracted their genomic DNA (BCSCs, non-BCSCs, NBBCs and non-NBBCs) at a single cell level and performed single-cell PCR for the TERT core promoter (Figure 1E). We analyzed twenty cells for each cell type. We found that a hypermutation of TERT promoter occurred both in BCSCs and non-BCSCs from all the detected patients (Figure 1F). The promoter mutation was a cytidine-to-thymidine transition (chr5, 1, 295, 228 C > T; hereafter termed C228T). The mutation rates of single cells were over 50% in BCSCs, whereas less than half in non-BCSCs (Figure 1G, Supplementary Table 2). No mutations were found in the other two normal cell types (NBBCs and non-NBBCs). We also checked the other frequent mutation (chr5, 1,295, 250 C > T; hereafter termed C250T) of the TERT core promoter as reported [17]. The mutation rates were hard to be detected in BCSCs and non-BCSCs (data not shown). Furthermore, four types of cells (BCSCs, non-BCSCs, NBBCs and non-NBBCs) were analyzed by exome sequencing to eliminate contamination by cancer cells (Table 1). In Table 1, we found there are some differences in terms of gene numbers, mutation sites and mutation types.

**Table 1:** Exome sequencing of BCSCs, non-BCSCs, NBBCs and non-NBBCs

**BCSCs**	**CHROM**	**POS**	**REF**	**ALT**	**QUAL**	**FILTER**	**Func**	**Gene**	**ExonicFunc**	**cytoband**
	4	1807894	G	T	7514.77	PASS	exonic	FGFR3	nonsynonymous SNV	4p16.3
	5	1255520	G	A	2957.77	PASS	exonic	TERT	nonsynonymous SNV	5p15.33
	12	25362217	A	G	113.9	PASS	UTR3	KRAS	.	12p12.1
	12	25368462	C	G	3109.77	PASS	exonic	KRAS	nonsynonymous SNV	12p12.1
	12	25388290	G	T	31.74	PASS	intronic	KRAS	.	12p12.1
	13	48919358	T	G	764.77	PASS	intronic	RB1	.	13q14.2
	13	48947469	G	T	539.77	PASS	intronic	RB1	.	13q14.2
	13	49027140	A	C	150.77	PASS	exonic	RB1	nonsynonymous SNV	13q14.2
	13	49051012	C	T	2518.77	PASS	intronic	RB1	.	13q14.2
	16	3781313	G	G	6740.77	PASS	exonic	CREBBP	nonsynonymous SNV	16p13.3
	16	3811556	A	G	15232.77	PASS	intronic	CREBBP	.	16p13.3
	16	3811596	C	T	8497.77	PASS	intronic	CREBBP	.	16p13.3
	22	41513175	T	C	281.77	PASS	intronic	EP300	.	22q13.2
	22	41537234	G	T	1184.77	PASS	intronic	EP300	.	22q13.2
	22	41543983	C	G	2608.77	PASS	intronic	EP300	.	22q13.2
	22	41551039	T	A	10641.77	PASS	exonic	EP300	synonymous SNV	22q13.2
	22	41553259	G	T	565.77	PASS	exonic	EP300	nonsynonymous SNV	22q13.2
**non-BCSCs**	**CHROM**	**POS**	**REF**	**ALT**	**QUAL**	**FILTER**	**Func**	**Gene**	**ExonicFunc**	**cytoband**
	4	1807894	G	T	19732.77	PASS	exonic	FGFR3	nonsynonymous SNV	4p16.3
	12	25362217	A	G	54.28	PASS	UTR3	KRAS	.	12p12.1
	12	25368462	C	A	279.78	PASS	exonic	KRAS	nonsynonymous SNV	12p12.1
	13	49051012	C	T	210.78	PASS	intronic	RB1	.	13q14.2
	16	3811556	A	G	5398.77	PASS	intronic	CREBBP	.	16p13.3
	16	3811596	C	T	2659.77	PASS	intronic	CREBBP	.	16p13.3
	16	3828847	C	CT	33.73	PASS	intronic	CREBBP	.	16p13.3
	22	41513175	T	C	153.77	PASS	intronic	EP300	.	22q13.2
	22	41537234	G	T	198.78	PASS	intronic	EP300	.	22q13.2
	22	41543983	C	G	636.77	PASS	intronic	EP300	.	22q13.2
	22	41553259	G	A	214.77	PASS	exonic	EP300	synonymous SNV	22q13.2
**NBBCs**	**CHROM**	**POS**	**REF**	**ALT**	**QUAL**	**FILTER**	**Func**	**Gene**	**ExonicFunc**	**cytoband**
	12	25368462	C	T	161.84	PASS	exonic	KRAS	.	12p12.1
	12	25388290	G	T	35.74	PASS	intronic	KRAS	synonymous SNV	12p12.1
	13	48919358	T	G	83.28	PASS	intronic	RB1	.	13q14.2
	13	48947469	G	T	112.9	PASS	intronic	RB1	.	13q14.2
	16	3811556	A	G	5016.77	PASS	intronic	CREBBP	.	16p13.3
	22	41537234	G	T	285.77	PASS	intronic	EP300	.	22q13.2
	22	41543983	C	G	671.77	PASS	intronic	EP300	.	22q13.2
**non-NBBCs**	**CHROM**	**POS**	**REF**	**ALT**	**QUAL**	**FILTER**	**Func**	**Gene**	**ExonicFunc**	**cytoband**
	12	25368462	C	T	161.84	PASS	exonic	KRAS	.	12p12.1
	12	25388290	G	T	35.74	PASS	intronic	KRAS	synonymous SNV	12p12.1
	13	48947469	G	T	112.9	PASS	intronic	RB1	.	13q14.2
	16	3811556	A	G	5016.77	PASS	intronic	CREBBP	.	16p13.3
	22	41543983	C	G	671.77	PASS	intronic	EP300	.	22q13.2

### The C228T mutation contributes to transformation of bladder cancer

**Figure 2:**
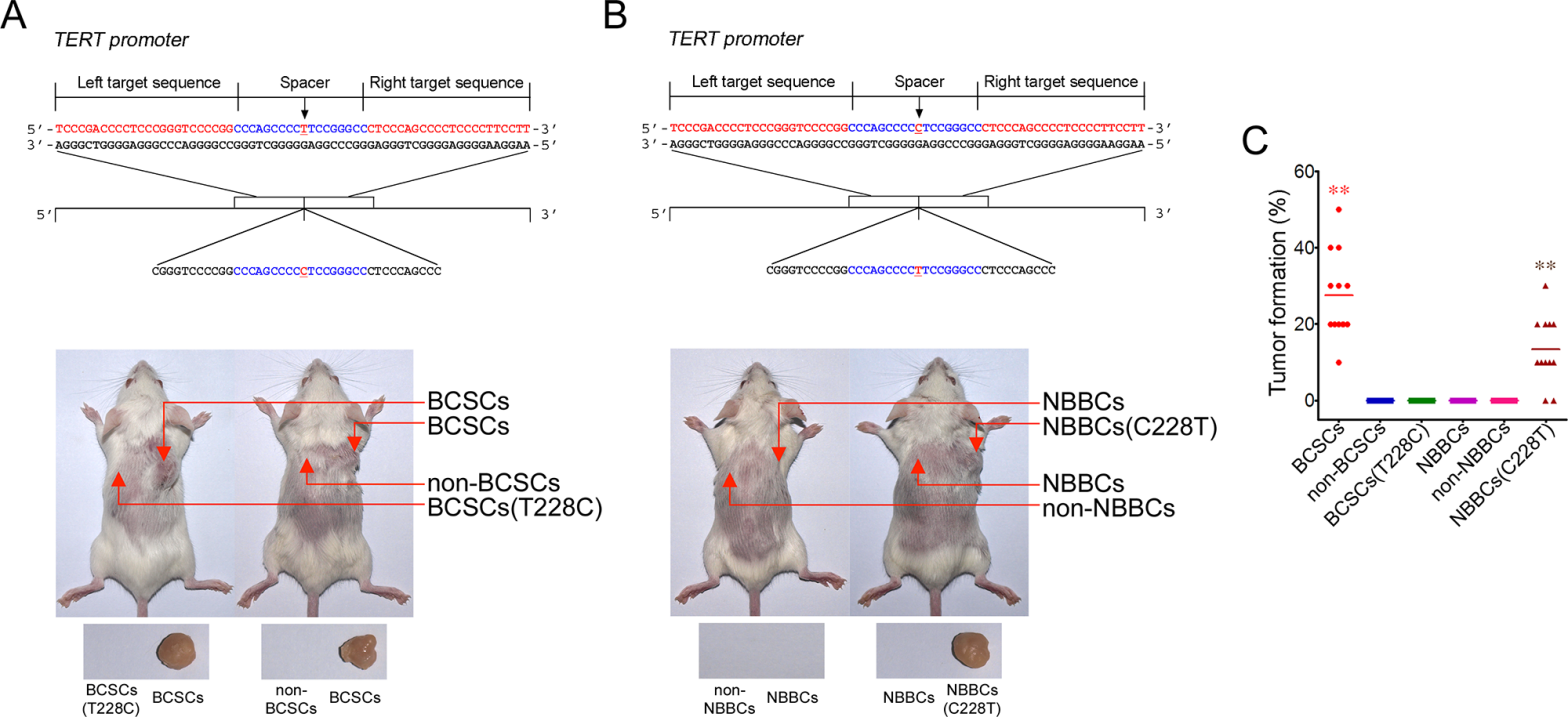
The C228T mutation contributes to tumorigenesis of bladder cancer **A.** BCSCs(T228C) was constructed by a TALEN approach. 100 cells for each subpopulations were injected intradermally into NOD/SCID mice followed by observing tumor formation in mice. Xenograft tumors denote with arrowheads. **B.** NBBCs(C228T) was established by the same TALEN approach and tumor formation was detected as above. **C.** Tumor formation rates of subpopulation cells were presented. **p < 0.01.

To explore the effect of the C228T mutation on the tumorigenesis of bladder cancer, we adopted a transcription activator-like effector nucleases (TALEN) approach to knock in the normal sequence (5′-CGGGTCCCCGGCCCAGCCCC CTCCGGGCCCTCCCAGCCC-3′) to replace the C228T mutation in BCSCs (called BCSCs(T228C)) (Figure 2A). Every one hundred cells, including BCSCs, non-BCSCs, BCSCs(T228C) were mixed with Matrigel (BD Biosciences) and injected intradermally into NOD/SCID mice, respectively. Of the 12 bladder cancer patient samples were successfully engrafted (Supplementary Table 3). The average tumor formation rate of BCSCs was 28.3% (from 10% to 50%), but no tumor appeared from BCSCs(T228C) xenografts (Figure 2C). These data suggest that restoration of the C228T mutation of TERT promoter can abolish tumor formation. To further verify these observations, we introduced the C228T mutation of TERT promoter in the NBBCs (termed NBBCs(C228T)) by the same TALEN approach (Figure 2B). Surprisingly, NBBCs(C228T) in 10 out of 12 bladder cancer patient samples, induced xenograft tumors *in vivo* (Figure 2B). The average tumor formation of NBBCs(C228T) is 15% (from 0% to 30%). However, no tumor formation from NBBCs and non-NBBCs (Figure 2B). Taken together, the C228T mutation contributes to transformation of bladder cancer.

### The C228T mutation causes TERT expression and telomerase activity in BCSCs

**Figure 3:**
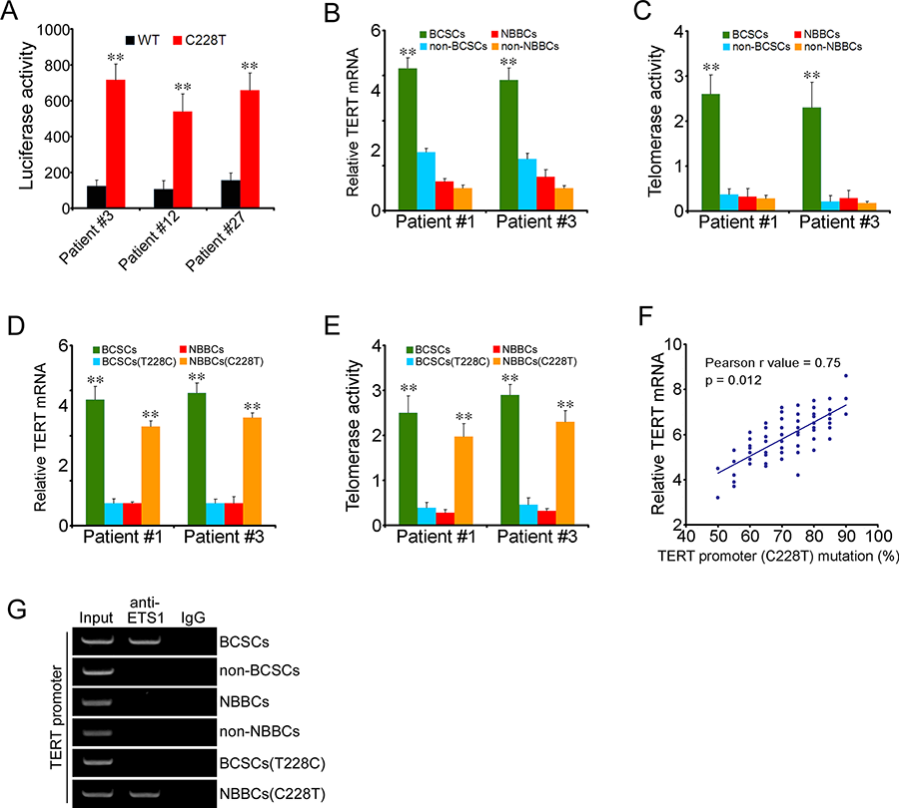
The C228T mutation causes TERT expression and telomerase activity **A.** Luciferase reporter assays for transcriptional activity of the TERT promoter. Patient#3, #12, #27 represented all the 60 bladder cancer samples. **B.** TERT mRNA levels in fractionated cells were detected by real-time PCR analysis. **C.** Telomerase activities in fractionated cells were detected by TRAP assays. **D, E.** TERT mRNA levels and telomerase activities were analyzed in BCSCs(T228C) and NBBCs(C228T). Patient#1 and #3 represented all the 60 bladder cancer samples. **F.** The correlation between the C228T mutation of TERT promoter and the TERT expression in BCSCs was analyzed from 60 bladder cancer patients (*p* = 0.012; *r* = 0.75, Pearson's correlation). **G.** Each subpopulation cells were harvested for ChIP assays with anti-ETS1 antibody, followed by PCR for TERT promoter. Data represent three different experiments. **p < 0.01.

We used a reporter assay system in which the relevant portion of the mutant or wild-type TERT core promoter was cloned upstream of the firefly luciferase gene. In comparison to the wild-type TERT promoter, the C228T mutation conferred approximately five-to sevenfold increased transcriptional activity for all the twelve patient's samples (Figure 3A). Thus, the TERT promoter mutation was capable of augmenting transcriptional activity. We examined TERT expression and telomerase activity in all sorted bladder cancer samples by real-time PCR and TRAP (telomeric repeat sequence amplification protocol) assays. We observed that TERT mRNA levels in BCSCs were much higher than in non-BCSCs (Figure 3B), while TERT mRNA remained low expression in NBBCs and non-NBBCs. Consistently, telomerase activity was higher in BCSCs than in non-BCSCs (Figure 3C). In contrast, telomerase activity kept at a basal level in NBBCs and non-NBBCs (Figure 3C). Interestingly, TERT mRNA levels and telomerase activity in BCSCs(T228C) returned back to a basal level (Figure 3D, 3E), similar to that of NBBCs. Importantly, TERT mRNA expression and telomerase activity in NBBCs(C228T) were significantly increased (Figure 3D, 3E), which were comparable to those of BCSCs. We next examined the correlation between the C228T mutation of TERT promoter and the TERT expression in sixty patient BCSCs. We found the TERT expression was significantly correlated with the C228T mutation of TERT promoter (*p* = 0.013; *r* = 0.75, Pearson's correlation) (Figure 3F).

The C228T mutation generated an identical 11-bp nucleotide stretch (5′-CCCCTTCCGGG-3′) harboring a consensus binding site for E-twenty-six (ETS) transcription factors (GGAA, reverse complement) in the TERT promoter region [17]. ETS transcription factors function either as transcriptional activators or repressors of numerous genes, involved in stem cell development, cell senescence and death, as well as tumorigenesis [20, 21]. ETS1 gene belongs to a member of the ETS transcription factor family. To determine whether the C228T mutation of TERT promoter binds to ETS1, we performed chromatin immunoprecipitation (ChIP) with an antibody against ETS1 from nuclear extracts of all sorted samples. We observed that anti-ETS1 antibody could enrich the TERT promoter in BCSCs (Figure 3G), but no enrichment in non-BCSCs, NBBCs or non-NBBCs. However, BCSCs(T228C) abolished the binding capacity (Figure 3G). Importantly, NBBCs(C228T) could enrich the TERT promoter to ETS1 gene. In summary, ETS1 was enriched to the TERT promoter in BCSCs and in NBBCs(C228T), which may contribute to TERT expression leading to telomerase activation. TERT was reported to be implicated in initiation and self-renewal of cancer stem cells 19. How TERT is involved in modulation of BCSC maintenance remains to be further investigated.

### TERT expression is consistent with clinical severity and prognosis of bladder cancer

**Figure 4:**
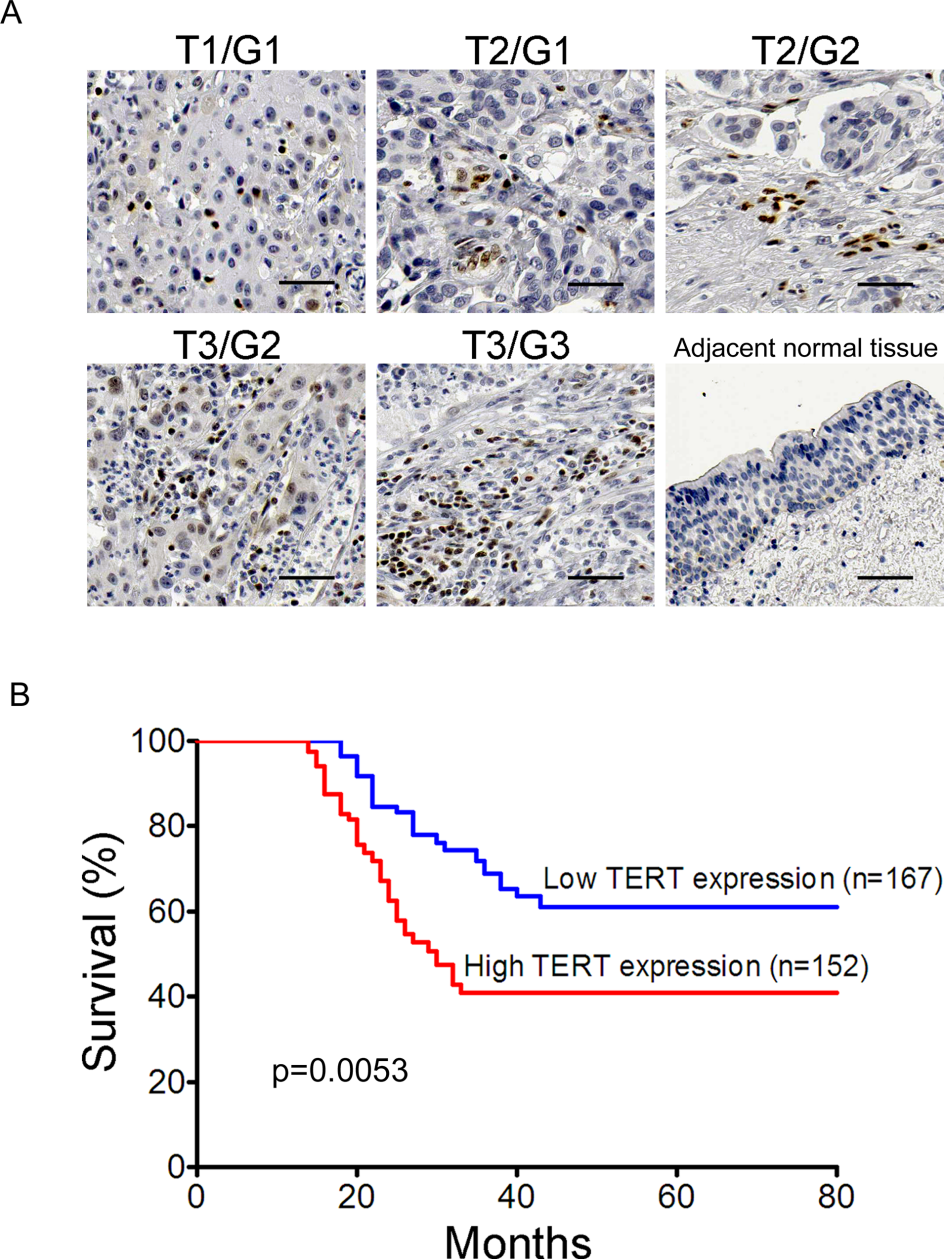
TERT is consistent with clinical severity and prognosis of bladder cancer **A.** TERT is associated with clinical severity. Tumor tissues and adjacent normal tissues were sectioned and detected through immunohistochemical staining with anti-TERT antibody. Sacle bar, 50 μm. **B.** TERT expression is correlated with prognosis of bladder cancer. Kaplan-Meier curve indicated the patients with higher levels of TERT expression had a worse overall survival (*p* = 0.0053).

To further investigate whether TERT is associated with clinical severity, we examined its expression in the bladder cancer patients through immunohistochemical staining. The intensity of TERT immunostaining was scored as follows: 1+, weak; 2+, moderate; and 3+, intense. Because tumors showed heterogeneous staining, the dominant pattern was used for scoring. The scores indicating percentage of positive tumor cells and staining intensity were multiplied to produce a weighed score for each case. Cases with weighed scores < 1 were defined as negative; cases with weighed scores > 2 were defined as strongly positive and those in between were defined as positive. We detected bladder tumor tissues from biopsies with classification of Grade (G1–3) and Stage (T1–4) among 319 bladder cancer patients. Low grades G1-G2 and low stages Ta-T1 were weakly stained (Figure 4A and Supplementary Table 4). Severe patients with high grade G3 and high stages T3-T4 showed intense staining of TERT. Normal bladder tissues were not stained as a negative control. The expression levels of TERT epitope correlated with pathological grades and tumor stages of bladder cancers.

We analyzed the relationship between expression of TERT and clinicopathological features in bladder cancer patients. The histologically high grade, deeply invasive and lymphatic-invasive bladder cancers demonstrated significantly higher expression of TERT than the low-grade, superficial, and nonlymphatic-invasive cancers (Supplementary Table 4). To further determine the association between TERT expression and prognosis, we completed 80-month follow-up of the above 319 bladder cancer patients with radical cystectomy. Intriguingly, the patients with high expression of TERT had significantly worse prognosis than patients with low expression (*P* = 0.0053) (Figure 4B).

## DISCUSSION

Telomerase is a ribonucleoprotein enzyme that is essential for the replication of chromosome termini in most eukaryotes. Telomerase is activated in cancer cells, and inactivated in normal somatic cells [22]. The TERT gene encodes the catalytic reverse transcriptase subunit of telomerase, which assembles the ribonucleoprotein enzyme complex that maintains telomere length. Recently, TERT promoter mutations were reported in several malignancies [17–19]. A recent report identified the high correlation of TERT promoter mutation and patient outcome in urothelial cancer [25]. They identified −124 C > T (corresponding to C228T) correlated with high TERT expression and telomerase activity. Our study mainly focused on the role of TERT mutation in bladder cancer stem cell regulation and tumorigenesis.

In our study, we demonstrated that C228T mutation within TERT promoter frequently occurred in BCSCs and this mutation contributed to tumor transformation of bladder cancer. Compared with non-BCSCs, C228T mutation rate was significantly higher in BCSCs and this is in consistence with the high TERT expression in stem cells but not in normal somatic cells. Therefore, high mutation rate in TERT promoter is a novel feature for BCSCs.

Very interestingly, C228T mutation at TERT promoter can efficiently transform normal NBBC into tumor-initiating cells. In parallel with oncogenes which result from driver mutations in protein-coding regions, the driver mutation in promoter region which can lead to oncogenesis can be considered as onco-promoters. Thus C228T mutation performs as a onco-promoter for bladder cancer. This result also indicated the potential origination of BSCS from mutated NBBC, which needs further investigation. Moreover, T228C mutation in BSCS can restore the normal TERT expression and abolish its tumor initiating capacity greatly. The TERT promoter acts as a gatekeeper controlling the transition between NBBC and BCSC. Collectively, the C228T mutation in TERT promoter may act as a driver to initiate bladder tumorigenesis from NBBC. In order to confirm this conclusion, genetically engineered mouse models (GEMM) should be employed in future study.

Our analysis from a cohort of 319 bladder cancer patients showed the high correlation between C228T mutation and bladder cancer development. Thus, C228T mutation can be selected as a useful novel prognosis marker for bladder cancer. Suppressing high TERT expression and telomerase activity is considered as a promising goal in bladder cancer therapy. Our research laid basis for this idea and novel therapeutics targeting BCSC for TERT inhibition needs to be developed.

## MATERIALS AND METHODS

### Antibodies, tissues and animals

The primary antibodies were phycoerythrin (PE)-conjugated anti-CD44 (BD PharMingen 550989), mouse anti-human TERT (Santa Cruz Biotechnology, US), mouse anti-human ETS1 (Santa Cruz Biotechnology, US). The Simple ChIP Enzymatic Chromatin IP Kit (#9003) was from Cell Signaling. Human bladder cancer tissues and normal human tissues were obtained from the Second People's Hospital of Shenzhen (Shenzhen, China) with informed consent, according to an IRB-approved protocol (Shenzhen IRB# 14). NOD/SCID mice were obtained from the Animal Center of the Chinese Academy of Medical Sciences, Beijing.

### Cell separation from bladder tumor tissues

The Institute of Biophysics Institutional Review Board approved the enrollment of human subjects. Tumor tissues of bladder cancer samples were dissociated in proteolytic (Accumax, Innovative Cell Technologies, Inc.), collagenolytic (200U Type I and 20U Type IV collagenase) (Sigma-Aldrich) and DNase enzymes at 37°C for 2 to 6 hours. Tissue cell suspensions were stained with phycoerythrin (PE)-conjugated anti-CD44 (BD PharMingen) antibody, and lineage mixture containing Cy7-PE-conjugated anti-CD45 (BD PharMingen), and biotin-conjugated anti-CD31 (eBioscience). Flow cytometry analysis and cell sorting was performed with a BD FACSAria (Becton Dickinson) cell-sorting system.

### Single-cell isolation and DNA extraction

Every step during the experiment was reduced to a strict minimum. With sufficient dispersion and cascade dilution of cells, single cells were randomly isolated from collagenase I and IV digested tumor or para-carcinoma tissues into PCR-ready tubes using an inverted microscope and a self-made mouth-controlled, fine hand-drawn micro-capillary pipetting system. The single cell isolation was visually confirmed by photograph under microscope. Afterward, single-cell DNA were performed using the REPLI-g Mini Kit (Qiagen) according to the manufacturer's instruction, using a no cell reaction as a negative control, and a reaction of human tissue genomic DNA as positive control.

### Identification and validation of TERT promoter mutations

Polymerase chain reaction (PCR) was performed on genomic DNA followed by direct sequencing on amplified PCR products on a subset of these tumor-adjacent normal bladder pairs to verify the individual TERT promoter mutations as well as on an additional 60 pairs for further confirmation for each sample. Forward primer: 5′-CAGCGCTGCCTGAAACTC-3′; Reverse primer: 5′-GTCCTGCCCCTTCACCTT-3′. Additionally the mutations were subcloned directly from tumor DNA using the TOPO (Invitrogen) cloning kit according to manufacturer's instructions followed by Sanger sequencing.

### TALEN approach to knock in mutations

The TALEN constructs were designed and generated according to a previous report (Zhang et al., 2011). The TERT(C228T) or TERT(T228C) TALEN vector was constructed to target exon 3 of human TERT gene promoter. The TALEN knockin sequences for TERT promoter were: TERT(C228T) TALEN vector: 5′-CGGGTCCCCGGCCCAGCCCCTTCCGGG CCCTCCCAGCCC-3′ and TERT(T228C) TALEN vector: 5′-CGGGTCCCCGGCCCAGCCCCCTCCGGGC CCTCCC

AGCCC-3′. TALEN constructs (1 μg) and ssOlig (1 μg) were co-transfected into BCSCs or NBBCs, respectively. After 48 h, 1 μg/ml puromycin was added into the medium for selection. Two days later, BCSCs or NBBCs were colonized on 96-well plate. After 10 days' selection, BCSCs or NBBCs colonies were harvested, and genomic DNA was extracted for PCR with identification primers: forward: 5′-CAGCGCTGCCTGAAACTC-3′, reverse: 5′-GTCCTGCCCCTTCACCTT-3′. PCR products were digested by NotI. The deletion of TERT promoter was confirmed by DNA sequencing. The positive clones of TALEN targeting were screened by puromycin selection and confirmed by DNA sequencing as described [23, 24].

### Transplantation of tissue cell suspensions into NOD/SCID mice

Tumor cells were suspended in Matrigel matrix mixed with media at a 1:1 ratio and were injected intradermally into the dorsal skin of NOD/SCID mice at age of 4 to 8 weeks by 31-gauge insulin syringes.

### Luciferase assays

Normal bladder basal stem cells were seeded at a density of 2 × 10 [4] cells in a 24-well format. Cells were cultured in DMEM/F12 media with B27, 20 ng/ml EGF, 20 ng/ml FGF and 4 mg/ml heparin (Life Technologies). Cells were transfected the following day using FuGENE 6 (Promega) with 2.25 μg of the TERT–luc promoter construct and 0.25 μg of pRL-TK (Promega), a control Renilla luciferase vector. 48 hours later, cells were lysed and luciferase activity was assayed with the Dual Luciferase Reporter (Promega) assay in a 96-well format according to manufacturer instructions. Experiments were performed in triplicate wells. Relative luciferase activity was calculated as the ratio of firefly to Renilla luciferase activity, to control for transfection efficiency. Control was the relative luciferase activity of cells transfected with promoterless reporter alone (pGL3-Basic).

### Telomerase activity assay

The TRAP (Telomeric repeat sequence amplification protocol) assay was carried out with the TRAPEZE Telomerase Detection Kit (Chemicon) following the manufacturer's protocol.

### Chromatin Immunoprecipitation (ChIP) assay

ChIP assays were performed with all sorted samples by using the anti-ETS1 antibody (Santa Cruz, sc-55581) with the Simple ChIP Enzymatic Chromatin IP Kit (#9003), following the standard protocol.

### Statistical analysis

The relationship between the staining levels of TERT and various clinicopathological factors was analyzed using the 2 or the Kruskal-Wallis test. Kaplan-Meier analysis was used to estimate the cumulative cause-specific survival rate, and the log-rank test was used to correlate differences in patient survival with staining intensity of TERT. In all statistical analyses, *p* values of 0.05 or less was considered to indicate statistical significance in two-sided test.

### Study approval

This study was started under the agreement of the patient and the institutional review board of the hospital approved the study, and informed consent was obtained according to the World Medical Association Declaration of Helsinki. All animal studies were permited by the IACUC of the Institute of Biophysics, Chinese Academy of Sciences and were conducted in compliance with its recommendations. All human studies were reviewed and approved by the IRB of Institute of Biophysics, Chinese Academy of Sciences, and written informed consent was provided according to the World Medical Association Declaration of Helsinki.

## SUPPLEMENTARY TABLES


