# Supplementary material for: The C228T mutation of TERT promoter frequently occurs in bladder cancer stem cells and contributes to tumorigenesis of bladder cancer
Source: Oncotarget. 2015 Jun 17;6(23):19542–51. doi: 10.18632/oncotarget.4295 (PMC4637304; doi:10.18632/oncotarget.4295)
Supplement: Supplementary file 1 [file oncotarget-06-19542-s001.pdf]

SUPPLEMENTARY TABLE LEGENDS

Supplementary Table S1. Characteristics of bladder cancer patients

Patient No.	Sex	Age	Stage	Grade	Superficial/ Invasive	Primary/ Relapsed
**1**	M	68	T3	2	Invasive	Primary
**2**	M	52	T2	3	Invasive	Relapsed
**3**	M	64	T3	2	Invasive	Primary
**4**	M	73	T2	3	Invasive	Primary
**5**	M	57	T2	2	Invasive	Primary
**6**	M	40	T3	2	Invasive	Primary
**7**	M	71	T4	2	Invasive	Primary
**8**	M	65	T4	1	Invasive	Primary
**9**	M	82	T3	3	Invasive	Primary
**10**	M	70	T4	3	Invasive	Primary
**11**	M	81	T3	3	Invasive	Primary
**12**	M	57	T4	2	Invasive	Primary
**13**	M	48	T3	2	Invasive	Primary
**14**	F	69	T2	1	Invasive	Primary
**15**	M	66	T3	2	Invasive	Primary
**16**	F	57	T3	3	Invasive	Primary
**17**	M	64	T2	3	Invasive	Primary
**18**	M	80	T3	2	Invasive	Relapsed
**19**	M	56	T2	2	Invasive	Primary
**20**	M	83	T3	2	Invasive	Primary
**21**	M	62	T3	3	Invasive	Primary
**22**	M	60	T2	2	Invasive	Primary
**23**	M	57	T2	2	Invasive	Relapsed
**24**	F	64	T2	3	Invasive	Primary
**25**	M	59	T3	1	Invasive	Primary
**26**	M	52	T3	2	Invasive	Primary
**27**	F	83	T3	1	Invasive	Primary
**28**	M	49	T2	1	Invasive	Primary
**29**	M	55	T3	2	Invasive	Primary
**30**	M	68	T3	2	Invasive	Primary
**31**	M	65	T3	3	Invasive	Relapsed
**32**	M	73	T2	2	Invasive	Primary

(*Continued*)

Patient No.	Sex	Age	Stage	Grade	Superficial/ Invasive	Primary/ Relapsed
**33**	M	47	T3	2	Invasive	Relapsed
**34**	M	83	T3	3	Invasive	Primary
**35**	M	78	T4	3	Invasive	Primary
**36**	M	65	T3	3	Invasive	Primary
**37**	F	53	T3	3	Invasive	Primary
**38**	M	82	T2	2	Invasive	Primary
**39**	M	65	T2	2	Invasive	Primary
**40**	M	66	T3	2	Invasive	Primary
**41**	M	53	T3	3	Invasive	Primary
**42**	M	57	T3	3	Invasive	Primary
**43**	M	64	T2	2	Invasive	Primary
**44**	M	75	T4	2	Invasive	Primary
**45**	F	76	T4	3	Invasive	Primary
**46**	M	69	T2	2	Invasive	Primary
**47**	M	56	T4	2	Invasive	Primary
**48**	F	73	T2	3	Invasive	Primary
**49**	F	73	T3	3	Invasive	Primary
**50**	M	65	T2	3	Invasive	Primary
**51**	M	58	T2	2	Invasive	Primary
**52**	M	79	T4	2	Invasive	Primary
**53**	F	80	T3	2	Invasive	Primary
**54**	M	57	T4	3	Invasive	Primary
**55**	M	66	T3	2	Invasive	Primary
**56**	M	54	T2	2	Invasive	Primary
**57**	M	56	T2	2	Invasive	Primary
**58**	M	60	T2	3	Invasive	Primary
**59**	M	78	T4	3	Invasive	Relapsed
**60**	M	65	T4	3	Invasive	Relapsed

Supplementary Table S2. TERT promoter mutations in subpopulation cell types of bladder cancer patients

Patient No.	BCSCs		non-BCSCs		NBBCs		non-NBBCs	
	C228T	C228C	C228T	C228C	C228T	C228C	C228T	C228C
1	13	7	9	11	0	20	0	20
2	17	3	12	8	0	20	0	20
3	14	6	7	13	0	20	0	20
4	16	4	10	10	0	20	0	20
5	14	6	5	15	0	20	0	20
6	17	3	12	8	0	20	0	20
7	15	5	6	14	0	20	0	20
8	11	9	3	17	0	20	0	20
9	16	4	12	8	0	20	0	20
10	13	7	10	10	0	20	0	20
11	16	4	11	9	0	20	0	20
12	16	4	13	7	0	20	0	20
13	15	5	13	7	0	20	0	20
14	13	7	12	8	0	20	0	20
15	16	4	13	7	0	20	0	20
16	15	5	12	8	0	20	0	20
17	12	8	10	10	0	20	0	20
18	18	2	14	6	0	20	0	20
19	15	5	12	8	0	20	0	20
20	16	4	10	10	0	20	0	20
21	17	3	11	9	0	20	0	20
22	15	5	12	8	0	20	0	20
23	13	7	10	10	0	20	0	20
24	12	8	9	11	0	20	0	20
25	11	9	8	12	0	20	0	20
26	14	6	10	10	0	20	0	20
27	18	2	16	4	0	20	0	20
28	14	6	7	13	0	20	0	20
29	16	4	12	8	0	20	0	20
30	13	7	3	17	0	20	0	20
31	15	5	12	8	0	20	0	20
32	15	5	7	13	0	20	0	20
33	13	7	10	10	0	20	0	20

(*Continued*)

Patient No.	BCSCs		non-BCSCs		NBBCs		non-NBBCs	
	C228T	C228C	C228T	C228C	C228T	C228C	C228T	C228C
34	17	3	11	9	0	20	0	20
35	15	5	12	8	0	20	0	20
36	16	4	10	10	0	20	0	20
37	12	8	5	15	0	20	0	20
38	16	4	11	9	0	20	0	20
39	11	9	10	10	0	20	0	20
40	17	3	12	8	0	20	0	20
41	14	6	10	10	0	20	0	20
42	17	3	11	9	0	20	0	20
43	12	8	9	11	0	20	0	20
44	13	7	5	15	0	20	0	20
45	18	2	13	7	0	20	0	20
46	14	6	9	11	0	20	0	20
47	17	3	12	8	0	20	0	20
48	12	8	9	11	0	20	0	20
49	15	5	12	8	0	20	0	20
50	16	4	11	9	0	20	0	20
51	12	8	7	13	0	20	0	20
52	17	3	15	5	0	20	0	20
53	13	7	12	8	0	20	0	20
54	14	6	11	9	0	20	0	20
55	12	8	10	10	0	20	0	20
56	13	7	9	11	0	20	0	20
57	11	9	8	12	0	20	0	20
58	14	6	9	11	0	20	0	20
59	15	5	10	10	0	20	0	20
60	14	6	7	13	0	20	0	20

Supplementary Table S3. Tumor formation rates from xenografted mice of subpopulation cell types of bladder cancer samples

Patient No.	100 cells tumor formation rate (%)					
	BCSCs	non-BCSCs	BCSCs (T228C)	NBBCs	non-NBBCs	NBBCs (C228T)
1	30	0	0	0	0	20
3	20	0	0	0	0	10
7	40	0	0	0	0	30
12	20	0	0	0	0	20
15	20	0	0	0	0	0
22	50	0	0	0	0	20
27	30	0	0	0	0	10
33	30	0	0	0	0	20
36	40	0	0	0	0	30
42	10	0	0	0	0	0
45	30	0	0	0	0	10
56	20	0	0	0	0	10

Supplementary Table S4. Relationship between the expression levels of TERT and clinicopathological features of human bladder cancers

			Expression of TERT		
**Total**		**Patients**	**High**	**Low**	***p***
Age	Mean	60.3	65.2	57.9	0.352
Sex	Male	237	109	128	0.428
	Female	82	37	45	
Tumor stage	Ta,Tis,T1	186	69	117	<0.001
	T2	59	38	21	
	T3	45	32	13	
	T4	29	23	6	
Grade	G1 or 2	142	54	88	<0.001
	G3	177	125	52	
Configuration	Papillary	46	24	22	0.708
	Nonpapillary	273	153	120	
Number of tumors	Solitary	252	136	116	0.082
	Multiple	67	24	43	
Lymphatic invasion	Negative	174	45	129	<0.001
	Positive	127	85	42	
	Unknown	18	11	7	
